# Location of Cerebral Microbleeds And Their Association with Carotid Intima-media Thickness: A Community-based Study

**DOI:** 10.1038/s41598-017-12176-y

**Published:** 2017-09-21

**Authors:** Chih-Ping Chung, Kun-Hsien Chou, Wei-Ta Chen, Li-Kuo Liu, Wei-Ju Lee, An-Chun Huang, Liang-Kung Chen, Ching-Po Lin, Pei-Ning Wang

**Affiliations:** 1 Department of Neurology, National Yang Ming University, Taipei City, Taiwan; 2 Institute of Brain Science, School of Medicine, National Yang Ming University, Taipei City, Taiwan; 3 Institute of Neuroscience, National Yang Ming University, Taipei City, Taiwan; 4 Aging and Health Research Center, National Yang Ming University, Taipei City, Taiwan; 5 Brain Research Center, National Yang Ming University, Taipei City, Taiwan; 6 Department of Neurology, Taipei Veterans General Hospital, Taipei City, Taiwan; 7 Center for Geriatric and Gerontology, Taipei Veterans General Hospital, Taipei City, Taiwan; 8 Department of Family Medicine, Taipei Veterans General Hospital Yuanshan Branch, Taipei City, Yi-Lan Taiwan

## Abstract

To assess whether high cerebral microbleeds (CMBs) are associated with carotid intima-media thickness (CIMT), a marker of systemic atherosclerosis, we cross-sectionally evaluated participants from a community-based study, the I-Lan Longitudinal Aging Study. The participants’ demographics and cardiovascular risk factors were determined by questionnaire and/or laboratory measurements. CIMT was measured by ultrasonography. CMBs were assessed by susceptibility-weighted-imaging on 3 T MRI. Of the 962 subjects [62.5(8.6) years, 44.2% men] included, CMBs were found in 134(14.0%) subjects. Among the subjects with identified CMB’s, 85(63.4%) had deep or infratentorial (DI) and 49(36.6%) had strictly lobar(SL) CMBs. After the results were adjusted for age and sex, the analysis revealed that hypertension, hyperlipidemia, obesity, and higher triglyceride levels correlated with DI but not SL CMBs. The subjects with DI CMBs also had a higher mean CIMT and higher prevalence of top quartile CIMT. The multivariate analysis demonstrated that high CIMT (top quartile) significantly predicted the presence of DI CMBs (odds ratio = 2.1; 95% confidence interval = 1.3–3.4; *P* = 0.004), independent of age, sex, cardiovascular risk factors, and other cerebral small vessel diseases, lacune, and white matter hyperintensity. There was no association between CIMT and SL CMBs. Our results support that there are distinct pathogenesis in DI and SL CMBs.

## Introduction

Lacunes, white matter hyperintensity (WMH), and cerebral microbleeds (CMBs) are the three main neuroimaging characteristics of Cerebral small vessel diseases (CSVDs)^1,2^. CMBs are small hemorrhages that appear as well-demarcated, hypointense, and rounded lesions on magnetic resonance imaging (MRI) sequences, and are sensitive to magnetic susceptibility^3^. Many studies have found that the presence of CMBs is associated with an increased risk of both ischemic and hemorrhagic strokes, mortality, and cognitive impairment^4–8^. Although their clinical significance, the mechanisms behind the formation of CMBs remain an active field of research.

CMBs that occur in different locations have distinct manifestations^3,9–11^. The prevalence of both types of CMBs increases with age. However, deep or infratentorial (DI) and strictly lobar (SL) CMBs are correlated with hypertension and APOE ɛ4 genotype, respectively^3,9,10^. Atherosclerosis is a systemic, chronic disorder that usually involves multiple vascular territories including the carotid, coronary, intracranial, and other peripheral arteries^11^. To elucidate the mechanisms of CMBs, previous studies have investigated the relationship of CMBs to markers of systemic atherosclerosis, such as the cardio-ankle vascular index, or carotid intima-media thickness (CIMT)^12,13^. However, these studies involved small patient populations and only investigated patients with a history of ischemic stroke. The association between CMBs and atherosclerosis might be confounded in stroke patients who already have advanced vascular diseases (silent or major infarcts, lacunes, advanced white matter hyperintensity, additional intracranial hematoma, brain atrophy, etc). In addition, the mechanisms and distributions of CMBs in stroke patients might be different than those in the general population.

CIMT measured by B-mode ultrasound has been the most widely used noninvasive imaging method to assess systemic atherosclerosis^11,14^. In the present study, we aimed to evaluate the relationship between CMBs and systemic atherosclerosis assessed by CIMT in a community-based, non-stroke population. We hypothesized that in the general population (1) CMBs are associated with atherosclerosis reflected by a greater degree of CIMT and (2) that the correlations differ according to the location of the CMBs.

## Results

### Study Population and Demographics

From the initial group of 989 subjects, two subjects with depression revealed by CES-D and nine subjects with an incidentally found brain tumor on MRI were excluded. There were also 16 subjects with imaging artifacts due to head motion. Thus, a total of 962 subjects were included in the present study with an age (mean ± SD, range) of 62.5 ± 8.6, 50.0–87.7 years old. Four-hundred and twenty-five (44.2%) of the subjects were men. The prevalence of cardiovascular risk factors in the population was as follows: hypertension, 37.0%; DM, 13.5%; hyperlipidemia, 6.0%; cigarette smoking habit, 26.1%; and obesity 5.5%. There were 50 subjects (5.2%) with a medical history of CAD and 18 subjects (2.9%) with CKD. The mean CIMT was 0.69 (SD: 0.13) mm; the CIMT values of the 25^th^, 50^th^, and 75^th^ percentile were 0.60, 0.70, and 0.75 mm respectively.

### Evaluation of CMBs and Other CSVDs (Lacunes and WMH)

CMBs were found in 134 (14.0%) subjects; among them, 61% had one CMB, 19% had two CMBs, and 18% had four or more CMBs. The majority of CMBs were found in the deep region. Among the subjects with CMB(s), 85 subjects (63.4%) had DI CMBs (with or without lobar CMBs) and 49 subjects (36.6%) had SL CMBs. The anatomic distribution of CMBs in our population showed BG 55 subjects (5.7%), thalamus 25 (2.6%), other deep regions 15 (1.6%), brainstem 17 (1.8%), cerebellum 13 (1.4%), frontal lobe 28 (2.9%), parietal lobe 19 (2.0%), temporal lobe 18 (1.9%), and occipital lobe 24 (2.5%). There was no CMB found in the regions of the internal capsule, external capsule, or corpus callosum.

Assessment of other CSVDs revealed that 45 (4.6%) subjects had more than one lacune and 151 (15.7%) subjects had moderate to severe WMH (Fazekas scale score of 2–3).

### Risk Factors for the Presence of Overall, DI and SL CMBs

The risk factor evaluation for the presence of overall, DI, and SL CMBs are shown in Table 1. The subjects with the presence of overall CMBs (without regard to CMBs location) were older. There was no difference in the sex distribution between the subjects with and without CMBs. After the data were adjusted for age and sex, the results revealed that subjects with CMBs had a higher prevalence of CAD, a higher number of lacunes, and more severe WMH. There was a trend toward an association with the presence of CMBs and a higher mean CIMT. When compared to subjects without CMBs, the prevalence of top quartile CIMT was significant higher in subjects with CMBs, after adjusting for age and sex.

**Table 1:** Variables Comparisons between Subjects with and without Cerebral Microbleeds.

	Overall			Deep/infratentorial CMBs			Strictly lobar CMBs		
	+(n = 134)	−(n = 828)	*p*	+(n = 85)	−(n = 877)	*p*	+(n = 49)	−(n = 913)	*p*
Age, years, mean (SD)	66.41 (9.66)	61.81 (8.18)	<0.001	66.89 (10.02)	62.03 (8.23)	<0.001	65.32 (8.62)	62.31 (8.53)	0.015
Sex, men, n (%)	61 (44.9)	364 (44.1)	0.926	40 (47.1)	385 (43.9)	0.648	20 (40.8)	405 (44.4)	0.660
Age and sex-adjusted									
Hypertension, n (%)	63 (46.3)	294 (35.6)	0.270	46 (54.1)	311 (35.5)	0.021	16 (32.7)	341 (37.3)	0.216
DM, n (%)	21 (15.4)	108 (13.1)	0.891	13 (15.3)	116 (13.2)	0.828	8 (16.3)	121 (13.3)	0.810
Hyperlipidemia, n (%)	14 (10.3)	44 (5.3)	0.111	11 (12.9)	47 (5.4)	0.032	2 (4.1)	56 (6.1)	0.423
Cigarette Smoking, n (%)	31 (22.8)	219 (26.5)	0.207	22 (25.8)	228 (26.0)	0.768	9 (18.3)	241 (26.4)	0.156
Obesity, n (%)	10 (7.4)	43 (5.2)	0.226	9 (10.6)	44 (5.0)	0.019	1 (2.0)	52 (5.7)	0.303
Coronary artery disease, n (%)	13 (9.6)	37 (4.5)	0.020	11 (12.9)	39 (4.4)	0.003	2 (4.1)	48 (5.3)	1.000
Chronic kidney disease, n (%)	2 (1.5)	12 (1.5)	0.572	2 (2.4)	12 (1.4)	0.914	0	14 (1.5)	0.999
Systolic BP*, mmHg, mean (SD)	131.4 (18.3)	127.8 (12.6)	0.224	132.4 (16.9)	127.9 (16.5)	0.206	129.0 (20.6)	128.3 (16.4)	0.993
Diastolic BP*, mmHg, mean (SD)	79.7 (12.1)	78.6 (12.7)	0.816	81.1 (12.1)	78.5 (12.6)	0.294	76.7 (10.9)	78.9 (10.9)	0.222
Pulse pressure*, mmHg, mean (SD)	51.8 (12.1)	49.2 (10.3)	0.137	51.3 (11.5)	49.4 (10.5)	0.547	52.3 (13.5)	49.3 (10.4)	0.153
LDL^†^, mg/dl, mean (SD)	112.9 (28.0)	117.8 (31.6)	0.506	114.1 (28.7)	117.4 (31.4)	0.893	109.9 (26.6)	117.5 (31.4)	0.149
TG^†^, mg/dl, mean (SD)	128.3 (98.9)	119.6 (73.7)	0.125	136.2 (116.2)	119.3 (72.9)	0.037	114.1 (59.4)	121.2 (78.6)	0.655
HDL^†^, mg/dl, mean (SD)	54.9 (12.7)	56.1 (14.4)	0.647	53.6 (12.1)	56.1 (14.4)	0.239	57.1 (13.8)	55.8 (14.2)	0.508
Lacune >1, n (%)	26 (19.1)	19 (2.3)	<0.001	24 (28.2)	21 (2.4)	<0.001	2 (4.1)	43 (4.7)	0.583
Moderate to severe WMH, n (%)	65 (47.8)	86 (10.4)	<0.001	49 (57.6)	102 (11.6)	<0.001	16 (32.7)	135 (14.8)	0.018
Carotid intima-media thickness									
Mean value, mm, mean (SD)	0.73 (0.13)	0.68 (0.12)	0.097	0.74 (0.14)	0.68 (0.12)	0.049	0.71 (0.12)	0.69 (0.13)	0.825
Top quartile, n (%)	58 (43.0)	211 (26.0)	0.034	43 (51.2)	226 (26.2)	0.002	15 (30.6)	254 (28.3)	0.711
Antiplatelets, n (%)	8 (5.9)	30 (3.6)	0.733	5 (5.9)	33 (3.8)	0.856	3 (6.1)	35 (3.8)	0.685

*p value was further adjusted for antihypertensive medicines using. ^†^ *p* value was further adjusted for statins using.

Both DI and SL CMBs were associated with increased age, but the correlations with cardiovascular risk factors were different between the two types of CMBs. After adjusting for age and sex, we found that DI CMBs were associated with hypertension, hyperlipidemia, obesity, CAD and higher circulatory triglyceride. In contrast, SL CMBs did not correlate with any cardiovascular risk factors. Notably, almost all subjects with hyperlipidemia had taken Stains (55/58, 94.8%). The association between hyperlipidemia and CMBs had been adjusted for Statin use.

The subjects with DI CMBs had a higher number of lacunes and more severe WMH than subjects without DI CMBs. SL CMBs were associated with WMH severity but not the number of lacunes. The association with CIMT also differed between DI and SL CMBs. When compared with subjects without DI CMBs, subjects with DI CMBs had significantly higher CIMT and a higher prevalence of top quartile CIMT. There was no association between CIMT and SL CMBs.

There were 38 (4%) subjects taking antiplatelets; 33 aspirin (100 mg per day) and 5 ticlopidine (100 mg twice per day). No subject in our population was prescribed anticoagulants. There was no association between CMBs and antiplatelet usage in the present study.

### Risk Factors for Top Quartile CIMT

Since a higher CIMT was a stronger predictor for vascular events and more representative of systemic atherosclerosis^15^, we used the top quartile CIMT as a marker of atherosclerosis in our analyses. The risk factors for high CIMT are presented in Table 2. Subjects with older age and male sex were more likely to have a higher CIMT. After adjusting for age and sex, the top quartile of CIMT was associated with hypertension and obesity.

**Table 2:** Variables Comparisons between Subjects with the Top and Lower Three Quartiles of Carotid Intima-thickness.

	Top Quartile of CIMT (n = 240)	Lower Three Quartiles of CIMT (n = 722)	*p*
Age, years, mean (SD)	67.1 (8.8)	60.5 (7.7)	<0.001
Sex, men, n (%)	153 (63.8)	267 (37.0)	<0.001
Age and sex-adjusted			
Hypertension, n (%)	129 (53.8)	220 (30.5)	0.018
DM, n (%)	46 (19.2)	79 (10.9)	0.562
Hyperlipidemia, n (%)	21 (8.8)	36 (5.0)	0.545
Cigarette Smoking, n (%)	97 (40.4)	149 (20.6)	0.188
Obesity, n (%)	21 (8.8)	30 (4.2)	0.005
Systolic BP*, mmHg, mean (SD)	131.6 (16.8)	126.8 (16.3)	0.097
Diastolic BP*, mmHg, mean (SD)	80.5 (14.1)	78.0 (11.9)	0.268
Pulse pressure*, mmHg, mean (SD)	51.1 (12.9)	48.8 (9.5)	0.251
LDL^†^, mg/dl, mean (SD)	116.1 (31.1)	117.4 (31.3)	0.107
TG^†^, mg/dl, mean (SD)	121.3 (76.6)	120.0 (75.9)	0.837
HDL^†^, mg/dl, mean(SD)	53.6 (13.5)	56.9 (14.4)	0.192
Lacune >1, n (%)	18 (7.5)	27 (3.7)	0.609
Moderate to severe WMH, n (%)	59 (24.6)	90 (12.5)	0.887

*p value was further adjusted for antihypertensive medicines using. ^†^ *p* value was further adjusted for statins using.

### Multivariate Analyses of Associations between CIMT and CMBs

Table 3 reveals the results of the multivariate logistic regression analyses. A higher CIMT (top quartile) was associated with the presence of CMBs independent of age, sex, cardiovascular risk factors, CAD, and CKD (model 1). The association was still significant after further adjustment for other CSVDs, the presence of lacunes, and the WMH (model 2). When the CMBs locations were considered, the results showed that the correlation between CMBs and CIMT was significant only in DI but not in SL CMBs. A higher CIMT predicted the presence of DI CMBs with an Odds ratio (OR) of 2.5, independent of age, sex, cardiovascular risk factors, CAD, CKD, and other CSVDs. SL CMBs were not associated with CIMT.

**Table 3:** Multivariate Logistic Regression Analyses for the Relationship between Cerebral Microbleeds and Carotid Intima-media Thickness.

	Overall			Deep/infratentorial CMBs			Strictly lobar CMBs		
	OR	95% CI	*p*	OR	95% CI	*p*	OR	95% CI	*p*
Top quartile IMT versus lower three quartile IMT, model 1*	1.8	1.1–2.8	0.013	2.5	1.4–4.3	0.001	0.9	0.5–1.8	0.765
Top quartile IMT versus lower three quartile IMT, model 2^†^	1.6	1.0–2.3	0.037	2.1	1.3–3.4	0.004	0.9	0.5–1.8	0.921

*Adjusted for age, sex, cardiovascular risk factors (hypertension, diabetes mellitus, hyperlipidemia, cigarette smoking, and obesity), coronary artery disease, and chronic kidney disease. ^†^Adjusted for age, sex, cardiovascular risk factors (hypertension, diabetes mellitus, hyperlipidemia, cigarette smoking, and obesity), coronary artery disease, chronic kidney disease, and other cerebral small vessel diseases (lacune and white matter hyperintensity).

## Discussion

The main finding of this study was that the presence of CMBs correlated with a higher CIMT. We also found that the location of the CMBs determined the association between the CMBs and CIMT; DI, but not SL, CMBs were significantly associated with a higher CIMT, independent of age, sex, cardiovascular risk factors (hypertension, DM, hyperlipidemia, cigarette smoking, obesity, CAD and CKD), and other CSVDs.

Previous studies with smaller population have investigated the relationship between CMBs and atherosclerosis in patients with a history of stroke^12,13^. The present community-based study evaluated the relationship between CMBs and atherosclerosis in the general population. Increased IMT has been deemed to be representative of early stages of atherosclerosis, and is widely used as a marker of atherosclerosis^11,14^. Our results indicate that CMBs in the general population were associated with atherosclerosis. This could explain why in several studies, the presence of CMBs predicts a higher risk of future not only hemorrhagic but also ischemic stroke independent of age, sex, and cardiovascular risk factors^16^. Since our study population included community-dwelling, subjects without a history of stroke, we postulated that atherosclerosis might be involved in the pathophysiology of CMBs at an early stage of the disease.

A recent-published paper of the Framingham Offspring Study revealed similar CMBs location-determined association with atherosclerosis markers^17^. Their results showed that carotid stenosis ≥25% was associated with DI but not SL CMBs. However, this study did not find a significant correlation between baseline CIMT and CMBs. The controversial results may due to the different study population. The other important reason might be that this study directly used CIMT value but not stratified ones into analysis. We classified CIMT into top quartile and the lower three quartiles since CIMT may be the sequela of other etiology instead of atherosclerosis and a higher CIMT was a stronger predictor for vascular events and more representative of systemic atherosclerosis^15^.

Our results also showed that DI but not SL CMBs were associated with atherosclerosis, independent of age, sex and cardiovascular risk factors. DI but not SL CMB, correlated with a history of CAD and lacunes in our study, a notion that also supports this postulation. In our population, DI CMBs were correlated with several atherogenic risk factors such as hypertension, hyperlipidemia, obesity, and higher triglyceride levels (Table 1), which might explain why only DI but not SL CMBs were associated with atherosclerosis.

Endothelial dysfunction, involved in the early stage of atherosclerosis^18^, might lead to impaired cerebral microvascular autoregulation in response to elevated perfusion pressure (such as in elevated BP) and consequent microvascular rupture^19,20^. It is possible that endothelial dysfunction mediates the association between atherosclerosis and DI CMBs.

Several studies have reported an association between arterial stiffness and age-related CSVDs, and showed that the relation of arterial stiffness to CMBs was different between the patients with stroke and the general population^21–23^. Previous studies in stroke patients have demonstrated an association between CMBs and arterial stiffness^12,13,24^. We did not find a correlation between CMBs, both DI and SL, and pulse pressure, which result was consistent with a previous study of subjects without a history of cerebrovascular and cardiovascular diseases^23,25^. Arterial stiffness, the reduced capability of an artery to expand and contract in response to pressure changes, usually develops in the elderly and people with long-standing hypertension^26^. The relationship between atherosclerosis and arterial stiffness has been analyzed and discussed in several studies^26^. Their correlation has been shown more consistently in more advanced atherosclerosis^26–28^. During the early stage of atherosclerosis, a thickened IMT may maintain vascular circumferential stress and eliminate vascular injury and prevent subsequent vascular structural changes. More advanced atherosclerosis or arterial stiffness would develop when the adaptive response is surpassed by persisted or profound vascular injury^29,30^. Our results revealed a relationship between DI CMBs with higher CIMT, but not pulse pressure in a community-dwelling, general population without a history of stroke, and it might reflect the pathogenesis of DI CMBs at a prodromal or early stage of disease. Arterial stiffness might follow atherosclerosis and be involved in the pathophysiology of DI CMBs at a later stage of disease. We would need a future longitudinal follow-up study to validate this hypothesis.

There were a few limitations to our study. Carotid plaque area and volume are other atherosclerosis markers^31^ which we did not measure in the present study. We were concerned that the frequency and severity of plaque in the younger and healthy population in the present study would have been too low to achieve statistical power with those carotid measurements. Therefore, we chose CIMT as the marker of atherosclerosis. Notably, increased CIMT might not always represent atherosclerosis pathology, therefore, we used a higher level of CIMT (top quartile), which is more specific for atherosclerosis^15^. Since both increased IMT and DI CMBs are correlated with hypertension, their relationship might simply be contributed by different hypertensive effects on vessels^31,32^, though we have adjusted with hypertension into our multivariate analyses. A recent study explored the association between carotid plaque volume (total and the subcomponents) and CMBs in 72 patients (CMB prevalence 35.3%); the results showed that an increased volume of the fatty component was associated with the presence and number of CMBs. Their method would be considered in the future study with a larger population to validate the relationship between DI CMBs and systemic atherosclerosis^33^. We also lacked information of inflammatory biomarkers^34^ and endothelial function^16^ in our population which could have provided clues to the mechanisms and the associations between atherosclerosis and DI CMBs. Finally, a longitudinal follow-up study is needed to see if higher level of CIMT leads to new DI formation. This information would strengthen our postulation that atherosclerosis is a causal factor in the pathophysiology of DI CMBs.

In conclusion, the present study has provided further evidence that there are pathogenic differences between DI and SL CMBs.

## Methods

### Study population

The I-Lan Longitudinal Aging Study is a community-based aging cohort study in the I-Lan County of Taiwan that aimed to evaluate the interrelationship between the geriatric syndromes and brain structural abnormalities and explore predictors or associated factors for future disable, dementia or mortality in the geriatric population when they are at a prodromal or early stage of disease^35^. In brief, community-dwelling adults aged 50 years or older from Yuanshan Township in I-Lan County were invited to participate in the study. Any subject that having any contraindication to an MRI such as metal implants, having been institutionalized for any reason or having known neuropsychiatric diseases such as dementia, stroke, brain tumor, or major depression was excluded from the study. The whole study was approved by the Institutional Review Board of the National Yang Ming University, Taipei, Taiwan. All the participants provided informed consent and we have conducted the study in accordance with the relevant ethical guidelines and regulations.

### Demographics and Cardiovascular Risk Factors

A questionnaire was used to collect data regarding the demographics and medical history of the study subjects. The heights, weights, and resting blood pressures (BP) of the subjects were measured. Fasting serum lipid levels (total cholesterol, low density lipoprotein, high density lipoprotein and triglyceride), and blood urea nitrogen and creatinine levels were determined by a chemical analyzer (ADVIA 1900, Siemens, Malvern, PA, USA). The presence of cardiovascular risk factors including cigarette smoking was determined by patient history or laboratory investigation. Hypertension was defined as a self-report of a current antihypertensive medication prescription or as a measurement of SBP ≥140 mmHg or DBP ≥90 mmHg^36^. Diabetes mellitus (DM) was defined as a self-report of current DM treatment or a measurement of HgbA1c ≥6.5%^37^. Hyperlipidemia was recorded if there was a self-report of the use of a statin agent or a total blood cholesterol level ≥240 mg/dL^38^. Body mass index (BMI) was calculated as weight in kg/height in m^2^. Obesity was defined as BMI ≥30 (World Health Organization, 2014). Chronic kidney disease (CKD) was defined as an estimated glomerular filtration rate ≤60 mL/min/1.73m^39^. A history of coronary artery disease (CAD) was determined by the subjects’ self-reports.

### Carotid Ultrasonography

All of the subjects underwent ultrasound imaging of the bilateral common carotid arteries (CCAs) in longitudinal projections using an instrument (GE LOGIQ 400 PRO; GE, Cleveland, OH, USA) equipped with a high-resolution broadband width linear array transducer by one technician. Each subject’s neck was extended in a supine position, with his/her head turned 30-degree toward the opposite direction of measurement. Then, the CIMT was measured on the far wall of subject’s right and left distal CCA by long axis view, and an image was automatically taken during R wave in electrocardiography. After taking an image, an operator selected 2 cm width region between carotid bifurcation and CCA, and then mean values of each CIMT were calculated by automatic methods in ultrasonography machine. The average of the left and right carotid IMTs were measured for analyses. All measurements were performed by one technician who was unaware of the clinical characteristics of the subjects. Measurements were additionally taken on a separate visit in 20 random-sampled subjects, and the intra-rater k = 0.80 (95% confidence interval 0.77–0.89).

### Brain MRI Acquisition and CMBs Assessment

All of the participants underwent a brain MRI study at National Yang-Ming University, Taipei, Taiwan. Images were acquired on a 3 T Siemens MRI scanner (Siemens Magnetom Tim Trio, Erlangen, Germany) with a 12-channel head coil. An axial T2-weighted fluid attenuated inversion recovery (FLAIR) multi-shot turbo spin echo sequence with BLADE technique was acquired with the following parameters: repetition time (TR) = 9000 ms, echo time (TE) = 143 ms, inversion time = 2500 ms, flip angle = 130 degree, number of excitation = 1, echo train length = 35, matrix size = 320 * 320, field of view (FOV) = 220 * 220 mm^2^, 63 slices, bandwidth = 252 Hz/Px, voxel size = 0.69 * 0.69 * 2.0 mm^3^ without inter-slice gap and acquisition time = 7 minutes and 41 seconds. Three dimensional susceptibility-weighted imaging (SWI) was used to identify the CMB. A three dimensional SWI sequence was acquired with the following parameters: TR = 28 ms, TE = 21 ms, flip angle = 15 degree, matrix size = 256 * 224, FOV = 256 * 224 mm^2^, 88 slices, bandwidth = 120 Hz/Px, voxel size = 1.0 * 1.0 * 2.0 mm^3^ without inter-slice gap and acquisition time = 9 minutes and 13 seconds.

CMBs were defined as small, rounded or circular, well-defined hypointense lesions within the brain parenchyma with clear margins that were ≦10 mm in size on the SWI image^3,40^. Microbleed mimics such as vessels, calcification, partial volume, air-bone interfaces, and hemorrhages within or adjacent to an infarct were carefully excluded. We distinguised calcification by viewing T1-weighted MRI. CMB mimics showing low density on T1-weight MRI will be regarded as calcification. We used the Microbleed Anatomical Rating Scale to measure the presence, amount, and topographic distribution of the CMBs in each subject, which has been reported a good intra-rater and inter-rater reliability^40^. The microbleeds were classified according to whether they were located in the deep, infratentorial, or lobar categories. Lobar topography was determined according to Stark and Bradley^41^, and included the cortical and subcortical regions (including subcortical U fibers). The lobar CMBs were assessed in the fontal, parietal, temporal and occipital regions. The deep regions included the basal ganglia (BG), thalamus, internal capsule, external capsule, corpus callosum, and deep/periventricular white matter; the infratentorial regions included the brainstem and cerebellum. DPWM was defined as white matter adjacent to or within approximately 10 mm of the lateral ventricular margin. Images were displayed and viewed using the MRIcro software (version 1.40, Chris Rorden’s MRIcro) by one neurologist (Dr. Chung) who was blinded to the clinical data during the CMB assessment and analyses. CMBs in 20 random-sampled subjects’ images were evaluated again at a separate time, and the intra-rater k = 0.83 (95% confidence interval 0.79–0.90). We also re-assessed CMBs in 25 random-sampled subjects’ images by Dr. Chung and her well-trained assistant (Mr. Ching-Sern Yong). The inter-rater k was 0.82 (95% confidence interval 0.79–0.88).

The other manifestations of CSVDs, the numbers of lacunes, and the severity of WMH were also recorded in every subject by FLAIR-T2-weighted MRI. Lacunes are small CSF-containing cavities, ≤15 mm in diameter, located in the deep grey or white matter with adjacent white matter hyperintensity^1^. The severity of WMH was rated by the modified Fazekas scale^42^. The Fazekas scale scores are: 0 as no WMH, 1 as mild WMH, 2 as moderate WMH, and 3 as severe WMH.

### Statistical Analysis

The statistical analyses were performed with SAS software, version 9.1 (SAS Institute, Cary, NC, USA). For continuous numeric variables, the nonparametric Mann-Whitney tests were performed as appropriate for group comparisons. The χ2 test or Fisher’s exact test was performed for categorical variables. Univariate and multivariate logistic regression analyses were performed to investigate the risk factors of (1) the presence of overall, DI, and SL CMBs respectively and (2) the top quartile CIMT. The associations between CMBs and the top quartile CIMT were also evaluated by multivariate logistic regression analyses. The results were presented as odds ratios (ORs) with 95% confidence intervals (95% CIs).

### Data availability statement

The datasets generated during and/or analysed during the current study are available from the corresponding author on reasonable request.
